# The Impact of Cognitive Impairment on Resource Utilization During Medicare Home Health Care

**DOI:** 10.1093/geroni/igaa057.835

**Published:** 2020-12-16

**Authors:** Julia Burgdorf, Jennifer Wolff

**Affiliations:** Johns Hopkins Bloomberg School of Public Health, Baltimore, Maryland, United States

## Abstract

Older adults with cognitive impairment have unique care needs that often lead to greater levels of health care utilization. Prior work suggests that older adults with cognitive impairment access home health care at higher rates; yet, recent Medicare home health payment system revisions exclude patient cognitive status when determining risk adjustment. This research examines the relationship between patient cognitive status and resource utilization during Medicare home health care. We examine 1,217 (weighted n=2,134,620) community-dwelling older adults who received Medicare-funded home health between 2011-2016, using linked nationally representative survey data from the National Health and Aging Trends Study (NHATS), home health patient assessment data, Medicare claims data, and Medicare Provider of Services files. We use weighted, multivariable negative binomial regressions to model the relationship between patient dementia status and the expected number of total visits and number of each visit type (nursing, therapy, and aide) during home health. Models adjusted for patient sociodemographic characteristics and health and functional status during home health, as well as home health provider characteristics. Among Medicare home health patients, the presence of cognitive impairment during home health is associated with 2.87 additional total visits (p<0.001), 1.27 additional nursing visits (p<0.01), and 1.23 additional therapy visits (p=0.04) during the home health episode. Findings suggest that recent revisions to the Medicare home health payment system may disincentivize home health care for older adults with dementia and/or financially penalize home health providers whose patient populations have a greater dementia burden.

